# Supplementary material for: What risk do Brucella vaccines pose to humans? A systematic review of the scientific literature on occupational exposure
Source: PLoS Negl Trop Dis. 2024 Jan 8;18(1):e0011889. doi: 10.1371/journal.pntd.0011889 (PMC10830018; doi:10.1371/journal.pntd.0011889)
Supplement: Attachment — Submitted filename: Review comments_PNTD-D-23-00623.docx [file pntd.0011889.s001.docx]

**Review comments**

Manuscript ID: PNTD-D-23-00623

General comments

At the first look at the first half of the title, I thought the scope is to discuss the risk from milk shed by vaccinated animals. The risk from milk cannot be ignored when considered Brucella vaccines. Also, microbiological and immunological aspects should be included. The number of articles examined is rather small, which may suggest the low attention paid by brucellosis endemic countries. The approach of systematic review is just summarizing these literatures, and there is no original analysis conducted for meaningful summary or comparisons. Moreover, I remember that a large-scale outbreak of brucellosis associated with a vaccine production unit in China a few years ago, but it is not included. Such incidence may not be in a scientific literature, and due to the same reason, the authors might have missed several other such accidents/incidence.

In brucellosis endemic countries, as occupational exposure is always one of the most important risk factors, infections with wildtype Brucella are very common; which means the authors’ argument that sero-positivity is due to Brucella vaccine is flawed, though some or a few of them can be truly due to the exposure to the vaccine strain. The study lacked the investigation into the quantitative information on the actual use (relative usage) of different types of the vaccine and the cases. The authors argue that Rev1 is the most virulent vaccine but the quantitative or qualitative evidence is not enough and the statement is not convincing. The suitability of this article to PLoS NTD should be judged by the editor, but I am not convinced due to above reasons. However, the information on the risk of brucellosis vaccine to humans is still indeed scarce. I would like to encourage the authors to anyway improve the article to make it more informative.

Specific comments

Material and methods

Line 69: Please Italicize “Brucella”.

Results

Line 75-76: The sentence is not clear: “Eleven of these articles were identified by manual search of the references found in the full text articles reviewed, and one of them was included in the study”. Out of 12, 11 were found from manual search and 1 was by what? Manual search is also not clear.

Line 108: “Since current standard serologic assays cannot detect antibodies against RB51 infection”, please provide the reference here. What about animals?

Line 146: RB51?

Line 158: Having brucellosis – having had brucellosis, or was the prevalence 4.6%?

Line 169: Sero-positivity associated with higher levels of exposure to the vaccine strains: what is the level of exposure you mean? High level is the high degree of invasion?

Discussion

Line 177: How did you judge that Rev.1 seems to be the most virulent?

Line 183: Please use references to state about the incidence in manufacturing workers.

Line 190: which country Spink conducted the clinical trial?

Line 199: clinical trial in Greece – it was considered safe enough: please discuss why it was considered so by the authors at that time.

Line 230: Please provide the key findings clearly when you refer systematic reviews. What were the risk factors for example? Exposure to vaccine strains through vaccination to animals?

Line 239-241: Enormous number of vaccine doses administered to humans? In which countries? Please present the evidence.

P266: Here please remind which were effective measures.
