# Supplementary material for: What risk do Brucella vaccines pose to humans? A systematic review of the scientific literature on occupational exposure
Source: PLoS Negl Trop Dis. 2024 Jan 8;18(1):e0011889. doi: 10.1371/journal.pntd.0011889 (PMC10830018; doi:10.1371/journal.pntd.0011889)
Supplement: Attachment — Submitted filename: Response to Reviewers.doc [file pntd.0011889.s002.doc]

| **Date:** | Aug 28 2023 08:11AM |
| --- | --- |
| **To:** | "José Javier Solera Santos" josejavier.solera@uclm.es |
| **cc:** | "Manuel Vives-Soto" mvives@quironsalus.es, "Amparo Puerta-García" amparo.puerta@quironsalud.es, "Esteban Rodríguez-Sánchez" e.rodriguez@biofabri.es, "José Luis Pereira" jl.pereira@biofabri.es |
| **From:** | "PLOS Neglected Tropical Diseases" plosntds@plos.org |
| **Subject:** | Decision on your PLOS Neglected Tropical Diseases submission (PNTD-D-23-00623) |
| **Attachment(s):** | [Review comments_PNTD-D-23-00623.docx](https://www.editorialmanager.com/pntd/download.aspx?guid={da3f3291-47e8-45d7-9481-7a60e20396f7}&scheme=13&id=19850) |

Dear Dr. Solera Santos,
 
Thank you very much for submitting your manuscript "What risk do Brucella vaccines pose to humans? A systematic review of the scientific literature on occupational exposure." for consideration at PLOS Neglected Tropical Diseases. As with all papers reviewed by the journal, your manuscript was reviewed by members of the editorial board and by several independent reviewers. In light of the reviews (below this email), we would like to invite the resubmission of a significantly-revised version that takes into account the reviewers' comments.
 
Please respond to author queries, or explain why any changes will be not made

 
We cannot make any decision about publication until we have seen the revised manuscript and your response to the reviewers' comments. Your revised manuscript is also likely to be sent to reviewers for further evaluation.
 
When you are ready to resubmit, please upload the following:

[1] A letter containing a detailed list of your responses to the review comments and a description of the changes you have made in the manuscript. Please note while forming your response, if your article is accepted, you may have the opportunity to make the peer review history publicly available. The record will include editor decision letters (with reviews) and your responses to reviewer comments. If eligible, we will contact you to opt in or out.

[2] Two versions of the revised manuscript: one with either highlights or tracked changes denoting where the text has been changed; the other a clean version (uploaded as the manuscript file).

Important additional instructions are given below your reviewer comments.

Please prepare and submit your revised manuscript within 60 days. If you anticipate any delay, please let us know the expected resubmission date by replying to this email. Please note that revised manuscripts received after the 60-day due date may require evaluation and peer review similar to newly submitted manuscripts.
 
Thank you again for your submission. We hope that our editorial process has been constructive so far, and we welcome your feedback at any time. Please don't hesitate to contact us if you have any questions or comments.
 
Sincerely,

Georgios Pappas
Academic Editor
PLOS Neglected Tropical Diseases

Ana LTO Nascimento
Section Editor
PLOS Neglected Tropical Diseases

***********************

Please respond to author queries, or explain why any changes will be not made


Reviewer's Responses to Questions

**Key Review Criteria Required for Acceptance?**


As you describe the new analyses required for acceptance, please consider the following:


**Methods**


-Are the objectives of the study clearly articulated with a clear testable hypothesis stated?

-Is the study design appropriate to address the stated objectives?

-Is the population clearly described and appropriate for the hypothesis being tested?

-Is the sample size sufficient to ensure adequate power to address the hypothesis being tested?

-Were correct statistical analysis used to support conclusions?

-Are there concerns about ethical or regulatory requirements being met?


Reviewer #1: The methods used for the systematic review are correct.

Reviewer #2: I have copied my comments under Summary and General Comments

Reviewer #3: The objective should include the risk of brucellosis due to milk consumption contaminated with vaccine isolate and the mechanism (microbiological and immunological).
The study design is not appropriate to capture all the vaccine related incidence, as such events may not be published in a scientific peer-review journals. Background vaccine use information is necessary but lacking.
The number of articles is small.

--------------------

**Results**


-Does the analysis presented match the analysis plan?

-Are the results clearly and completely presented?

-Are the figures (Tables, Images) of sufficient quality for clarity?


Reviewer #1: (No Response)

Reviewer #2: I have copied my comments under Summary and General Comments

Reviewer #3: The results failed to show all the evidence of vaccine related brucellosis infections.
Table itself showed that the results are not representative of global vaccine related incidence. And they are not comparable or compared.

--------------------

**Conclusions**


-Are the conclusions supported by the data presented?

-Are the limitations of analysis clearly described?

-Do the authors discuss how these data can be helpful to advance our understanding of the topic under study?

-Is public health relevance addressed?


Reviewer #1: The conclusions are in general supported by the data presented, although some specific comments are provided below.

Reviewer #2: I have copied my comments under Summary and General Comments

Reviewer #3: No.

--------------------

**Editorial and Data Presentation Modifications?**


Use this section for editorial suggestions as well as relatively minor modifications of existing data that would enhance clarity. If the only modifications needed are minor and/or editorial, you may wish to recommend “Minor Revision” or “Accept”.

Reviewer #1: (No Response)

Reviewer #2: I have copied my comments under Summary and General Comments

Reviewer #3: (No Response)

--------------------

**Summary and General Comments**


Use this section to provide overall comments, discuss strengths/weaknesses of the study, novelty, significance, general execution and scholarship. You may also include additional comments for the author, including concerns about dual publication, research ethics, or publication ethics. If requesting major revision, please articulate the new experiments that are needed.

**Reviewer #1**:

**1) Lines 34-36. “Despite strict protection measures, a percentage of vaccine manufacturing workers developed a positive serology to the vaccine strain, which may have conferred immunity.”
COMMENT: Serological tests do not allow determining the Brucella species that elicited the antibody response. Moreover, cross-reactivity with other bacteria may lead to false positive results. In addition, please provide references supporting the protection conferred to humans by the accidental exposure to vaccine strains.**

**Answer**: We agree with the reviewer and appreciate his comments. So, we have deleted the phrase from de Abstract section and changed the Results seccion in the same sense.

**Change in the manuscript:**

ABSTRACT (lines 38-40): *“Besides, despite strict protection measures, a percentage of vaccine manufacturing workers developed positive Brucella serology without clinical infection.”*

RESULTS (lines 190-196): *“Although serology does not allow to distinguish between vaccine and wild strains, the possibility of contact with Brucella outside the factory was minimal, given the almost absence of cases in the region where the factory is located: <0.08 cases per 100,000 inhabitants / year in the local official registry (https://www.sergas.es/Saude-publica/Documents/105/BEG_XXV-1.pdf). The fact that none of the workers studied developed the disease could be explained by the lower virulence of both vaccine strains and / or by a smaller bacterial inoculum due to strict compliance with safety measures.”*

**2) While a systematic review of the relevant literature has been performed, some reports of human infection by vaccine Brucella strains seem to be missing. For example, an important outbreak that took place in China in 2019 should be mentioned (Pappas G, Clin Infect Dis. 2022 Nov 14;75(10):1845-1847; Baoshan L et al., Transbound Emerg Dis 2021 Mar;68(2):368-374.) We also recommend including relevant information provided in chapter 8 of the book Brucellosis Clinical and Laboratory Aspects (Young EJ, Corbe MJ, CRC Press, 1989).**

**Answer**: We thank the reviewer for pointing up this important outbreak of vaccine brucellosis in humans and have added this report to the review. Furthermore, following the reviewer's suggestion, the bibliographic search has been expanded with new algorithms and until September 30, 2023, managing to add 7 new articles into the study. The new search strategy is described in the "Material and methods" section and the number of articles selected is listed in the first paragraph of the "Results" section. Also, we have reviewed chapter 8 of the book “Brucellosis Clinical and Laboratory Aspects” (Young EJ, Corbe MJ, CRC Press, 1989) and the most relevant information has been included in the Introduction section [Reference number 9].

**Change in the manuscript:**

INTRODUCTION (lines 78-88): *”These strains are capable of establishing limited infection in livestock, mimicking the natural infection process by wild strains and thus conferring protection. However, these vaccines are not used in humans due to the high risk of developing acute brucellosis [4,7]; they are capable of infecting humans with occupational exposure via the oral, nasal, or conjunctival or genital routes, and by accidental needle inoculation [8,9]. Whatever the route of entry, the infection can be symptomatic or asymptomatic, and localized or systemic [9,10]. Serologic tests continue to be used to diagnose brucellosis, given the risk and difficulty involved in obtaining positive cultures [9,11]. However, serology cannot distinguish between vaccine and wild strains and current standard serologic assays cannot detect antibodies against RB51 infection [12]. The recent development of molecular techniques (PCR) has made it possible to distinguish vaccine from wild strains in animal and human samples [13].”*

MATERIAL AND METHODS (lines 107-117): *“The guidelines of the PRISMA statement (Preferred Reported Items for Systematic Reviews and Meta-Analyses) were formally adopted in this review [17]. The search was conducted on September 30, 2023, with no date or country restrictions, using the PubMed database. Next, titles, abstracts and full texts were independently analyzed by two investigators. Three search algorithms were applied consecutively: firstly “Brucella AND (Rev-1 OR Rev.1 OR S19 OR S.19 OR B19 OR B-19 OR RB51 OR A19 OR S2) AND Human[Mesh]”, secondly “Brucella AND Vaccine AND occupational AND Human[Mesh]”, and thirdly “("Laboratory Infection"[Mesh]) AND Brucella[Mesh]”.”*

RESULTS (lines *119-123*): *“The PRISMA Flow Diagram is shown in Fig 1. Nineteen articles were included from 185 records screened. Of the articles finally included, 12 were identified with the first algorithm, 3 with the second algorithm, 1 with the third algorithm, and 3 more from review of the reference lists. Table 1 lists the publications of brucellosis cases acquired through laboral exposure to vaccine strains.”*

The others changes can be visualized in the new Fig.1 and Table.1.

**3) Lines 31-32. In our opinion, exposure accidents during vaccine manufacturing should not be considered exceptional, as several cases have been described.**

**Answer**: We agree with the reviewer.

**Change in the manuscript:** The terms “were exceptional” has been changed by “were uncommon”:

ABSTRACT (lines 36-41): *“Although accidental exposure during vaccine manufacturing were uncommon, they could provoked large outbreaks through airborne spread with risk of spread to the neighboring population. Besides, despite strict protection measures, a percentage of vaccine manufacturing workers developed positive Brucella serology without clinical infection. The most frequent type of exposure with symptomatic infection was needle injury during vaccine administration.”*

4) **Lines 41-44. “Human brucellosis is a zoonosis with worldwide distribution. Although the number of cases of brucellosis may be decreasing in the world, the continued presence of the disease in some endemic areas and the potential use of Brucella species as an agent of bioterrorism, make brucellosis a major public health hazard with important sanitary and economic repercussions”.
COMMENT: As the apparent decrease of brucellosis cases may be due to subnotification, I suggest to use “reported cases” instead of “cases”.**

**Answer**: We agree with the reviewer.

**Change in the manuscript:** The term "the number of cases" has been changed to "t*he number of reported cases*".

INTRODUCTION (lines 65-71): *“Human brucellosis is a zoonosis with worldwide distribution. A recent study has estimated that there are at least 1.6 - 2.1 million new cases of human brucellosis each year [1]. Although the number of reported cases have decreased in more developed countries, the continued presence of the disease in some endemic areas, particularly in Eastern Europe, the Asia-Pacific, Central and South America and Africa, and the potential use of Brucella species as an agent of bioterrorism, make brucellosis a major public health hazard with important sanitary and economic repercussions [2].”*

**5) Lines 52-55. “However, these vaccines are not used in humans due to the high risk of developing acute brucellosis; they are capable of infecting humans with occupational exposure via the oral, nasal, conjunctival or genital routes, and by accidental needle inoculation”.
COMMENT: To our best knowledge, there are no reports of vaccine strain infection in humans through the genital route.**

**Answer**: Certainly the genital route cannot be included as an occupational exposure. We appreciate the correction and the term has been removed.

**Change in the manuscript:** The text has remained:

INTRODUCTION (lines 79-82): *“However, these vaccines are not used in humans due to the high risk of developing acute brucellosis [4,7]; they are capable of infecting humans with occupational exposure via the oral, nasal, or conjunctival routes, and by accidental needle inoculation [8,9].”*

**Reviewer #2**:

**In this study, the authors reviewed the risk of occupational exposure to the Brucellosis vaccine. The research question is valid and is of interest to the journal audience. However, the study does not appear to be well designed and conducted.**

**Although the authors report that they followed the PRISMA guidelines, it is surprising to note that 11 of the 12 studies finally selected for inclusion in the systematic review were not in the systematic search they conducted but rather were selected based on a manual search of references found in the full-text articles reviewed. This nullifies any of the benefits of the systematic search and incorporates bias into the search that the systematic review aims to avoid.**

**Answer**: We agree with the revier that our sentence was not clear enough and appreciate his comments. The bibliographic search has been expanded with new algorithms and until September 30, 2023, allowing to add 7 new articles into the study. The new search strategy is described in the "Material and methods" section and the number of articles selected is listed in the first paragraph of the "Results" section.

**Change in the manuscript:**

MATERIAL AND METHODS (lines 107-117): *“The guidelines of the PRISMA statement (Preferred Reported Items for Systematic Reviews and Meta-Analyses) were formally adopted in this review [17]. The search was conducted on September 30, 2023, with no date or country restrictions, using the PubMed database. Next, titles, abstracts and full texts were independently analyzed by two investigators. Three search algorithms were applied consecutively: firstly “Brucella AND (Rev-1 OR Rev.1 OR S19 OR S.19 OR B19 OR B-19 OR RB51 OR A19 OR S2) AND Human[Mesh]”, secondly “Brucella AND Vaccine AND occupational AND Human[Mesh]”, and thirdly “("Laboratory Infection"[Mesh]) AND Brucella[Mesh]”.”*

RESULTS (lines *119-123*): *“The PRISMA Flow Diagram is shown in Fig 1. Nineteen articles were included from 185 records screened. Of the articles finally included, 12 were identified with the first algorithm, 3 with the second algorithm, 1 with the third algorithm, and 3 more from review of the reference lists. Table 1 lists the publications of brucellosis cases acquired through laboral exposure to vaccine strains.”*

The others changes can be visualized in the new Fig.1 and Table.1*.*

**Secondly, the authors have a tendency to summarise information from the studies rather than comparing and contrasting or evaluating evidence and developing an argument. This is not acceptable, even in a simple critical review.**

**Answer**: We appreciate this outstanding observation and have redrafted the "RESULTS" section, classifying the studies into three subsections: safety accidents in vaccine factories, exposure during manufacturing, and exposure during vaccine administration. Within each section the evidence has been contrasted and an attempt has been made to develop a critical argument.

**Change in the manuscript:** The first paragraph of the "RESULTS" and “DISCUSSION” sections summarizes the new approach. In addition, Table 1 has been reorganized to bring together the studies of each subgroup.

RESULTS (lines 119-125): *“The PRISMA Flow Diagram is shown in Fig 1. Nineteen articles were included from 185 records screened. Of the articles finally included, 12 were identified with the first algorithm, 3 with the second algorithm, 1 with the third algorithm, and 3 more from review of the reference lists. Table 1 lists the publications of brucellosis cases acquired through laboral exposure to vaccine strains. These publications are summarized below in three subgroups: outbreaks after safety accidents in vaccine factories, risk for vaccine manufacturing workers, and risk during vaccine administration.”*

DISCUTION (lines 286-302): *“Our results show that there is a risk of occupational infection by Brucella vaccine strains, although the small number of symptomatic infections recorded compared to the enormous number of doses administered [https://www.coherentmarketinsights.com/market-insight/brucellosis-vaccines-market-5038], suggests that most of them are subclinical. Human can be infected through aerosol exposure and by mucosal and non-intact skin contact with live attenuated strains. Of the 5 vaccine strains currently used (Rev.1, S19, RB51, A19 and S2), Rev.1 seems to be the most virulent [7]. Prolonged contact with the pathogen, lack of information and instructions provided to the occupational groups exposed, and low adherence to personal protective equipment (PPE) in the work environment, appeared to be the main risk factors leading to infection by these vaccine strains [24,36,37,38]. Vaccine factories accidents are infrequent but can cause serious outbreaks due to aerial spread. On the other hand, despite strict protection measures, a percentage of vaccine manufacturing workers developed positive serology to the vaccine strain without symptomatic illness [23,24]. In fact, Buchanan et al. had observed in slaughterhouses that workers with positive serology showed a lower risk of acquiring brucellosis [39]. Among veterinarians and other vaccine administration workers, vaccine handling was the most reported source of exposure to Brucella. ”*

Changes can be visualized in the new Table.1.

**Thus, although the subject is worthy of investigation, the manuscript would have to be substantially improved before it can be published in PLOS Neglected Tropical Diseases.**

**Answer:** Finally, we appreciate the reviewer's criticism and hope to have substantially improved the article.

**Change in the manuscript:** The bibliographic search has been expanded and updated as of September 30, 2023 and all sections of the article have been substantially modified following the reviewers' instructions. The new article, table-1 and figure-1 are sent attached to this letter.

**Reviewer #3:**

**General comments: At the first look at the first half of the title, I thought the scope is to discuss the risk from milk shed by vaccinated animals.**

**Answer:** We thank the reviewer for this important observation. Indirect exposure to these Brucella vaccine strains outside the scope of their manufacture and administration to livestock is an extensive research topic beyond the scope of the present review. However, we have taken note of this observation and are considering to conduct a new research to address this issue.

**The risk from milk cannot be ignored when considered Brucella vaccines. Also, microbiological and immunological aspects should be included.**

**Answer**: Following the reviewer's instructions, we have included in the "DISCUSSION" section a paragraph on the three possible scenarios of indirect exposure to vaccine strains, also including microbiological and immunological aspects.

**Change in the manuscript:**

DISCUSSION (lines 323-356): *“On the other hand, indirect exposure to Brucella vaccine strains has been reported, including the assistance to livestick births and abortions, handling dairy products, and analyzing contaminated samples in clinical laboratories. As explained below, symptomatic cases were not due to exposure during vaccine manufacturing or administration. They were not included in this review since cases were not due to exposure during vaccine manufacturing or administration. In 1998, the CDC described the case of a stillborn calf, delivered by cesarean. The necropsy revealed that death was due to infection by the RB51 strain Brucella abortus [32]. The strain was isolated from placental and fetal lung tissue, as well as from the blood of the calf´s mother and was identified by molecular methods (PCR). The nine persons who participated in the procedures received post-exposure prophylaxis, and none developed brucellosis during the 6-month follow-up period. Besides, Brucella abortus RB51 and S19 is transiently excreted in the milk of vaccinated cattle and can survive throughout the manufacture and conservation processes of both fresh and ripened cheeses [43] which poses a risk of infection through ingestion. In this setting, in 2015 Osma et al. reported a serosurveillance study on 100 asymptomatic farmworkers employed in Khartoum, Sudan [44]. Ten of them tested seropositive, including 4 milkers with a positive blood culture, and only in one of them the S19 strain was identified by PCR, but milk ingestion could not be excluded. Later, in 2018, Cossaboom et al. reported a case of human brucellosis associated with the consumption of unpasteurized cow’s milk purchased from a dairy in Paradise, Texas, USA [45]. The CDC’s Bacterial Special Pathogens Branch (BSPB) confirmed the isolate as Brucella abortus vaccine strain RB51. Recently, in 2021, Sarmiento-Clemente et al. reported in Houston, USA, the first case of neurobrucellosis due to the vaccine strain RB51 [46]. The patient was an 18-year old Hispanic female who had consumed unpasteurized cheese brought from Mexico 1 month before onset of symptoms. The strain was isolated in blood cultures and identified by molecular methods in the Houston Health Department/Laboratory. The authors also mentioned that the CDC reported 3 confirmed cases in the United States of human infection by RB51 through consumption of raw milk. Finally, in regard to clinical laboratory exposure, the CDC conducted in 2007 a laboratory proficiency test to a total of 254 laboratories of USA and Canada with potential RB51 exposure [47]. Despite correct labeling of the samples, 916 laboratory workers did not handled the RB51 samples properly, including 679 (74%) with high-risk exposures and 237 (26%) with low-risk exposures, although no cases of brucellosis were reported. The authors highlight the need for routine adherence to recommended biosafety practices when working with infectious organisms.”*

**The number of articles examined is rather small, which may suggest the low attention paid by brucellosis endemic countries. The approach of systematic review is just summarizing these literatures, and there is no original analysis conducted for meaningful summary or comparisons. Moreover, I remember that a large-scale outbreak of brucellosis associated with a vaccine production unit in China a few years ago, but it is not included. Such incidence may not be in a scientific literature, and due to the same reason, the authors might have missed several other such accidents/incidence.**

**Answer**: We agree with the reviewer that the fact of having omitted this important epidemiological outbreak in China calls into question the validity of the algorithm use for literature searching. For this reason, this algorithm has been reformed, allowing us to locate this outbreak and others previously overlooked. Thus, 7 new articles have been added into the study. The new search strategy is described in the "Material and methods" section and the number of articles selected is listed in the first paragraph of the "Results" section. Moreover, we have been able to include in the study papers from some endemic countries such as Bosnia Herzegovina, Turkey, Brazil, India and China. Besides, we have redrafted the "RESULTS" section, classifying the studies into three subsections: safety accidents in vaccine factories, exposure during manufacturing, and exposure during vaccine administration. Within each section the evidence has been contrasted and an attempt has been made to develop a critical argument.

**Change in the manuscript:** The first paragraph of the "RESULTS" and “DISCUSSION” sections summarizes the new approach. In addition, Table 1 has been reorganized to bring together the studies of each subgroup.

RESULTS (lines 119-125): *“The PRISMA Flow Diagram is shown in Fig 1. Nineteen articles were included from 185 records screened. Of the articles finally included, 12 were identified with the first algorithm, 3 with the second algorithm, 1 with the third algorithm, and 3 more from review of the reference lists. Table 1 lists the publications of brucellosis cases acquired through laboral exposure to vaccine strains. These publications are summarized below in three subgroups: outbreaks after safety accidents in vaccine factories, risk for vaccine manufacturing workers, and risk during vaccine administration.”*

DISCUSSION (lines 286-302): *“Our results show that there is a risk of occupational infection by Brucella vaccine strains, although the small number of symptomatic infections recorded compared to the enormous number of doses administered [https://www.coherentmarketinsights.com/market-insight/brucellosis-vaccines-market-5038], suggests that most of them are subclinical. Human can be infected through aerosol exposure and by mucosal and non-intact skin contact with live attenuated strains. Of the 5 vaccine strains currently used (Rev.1, S19, RB51, A19 and S2), Rev.1 seems to be the most virulent [7]. Prolonged contact with the pathogen, lack of information and instructions provided to the occupational groups exposed, and low adherence to personal protective equipment (PPE) in the work environment, appeared to be the main risk factors leading to infection by these vaccine strains [24,36,37,38]. Vaccine factories accidents are infrequent but can cause serious outbreaks due to aerial spread. On the other hand, despite strict protection measures, a percentage of vaccine manufacturing workers developed positive serology to the vaccine strain without symptomatic illness [23,24]. In fact, Buchanan et al. had observed in slaughterhouses that workers with positive serology showed a lower risk of acquiring brucellosis [39]. Among veterinarians and other vaccine administration workers, vaccine handling was the most reported source of exposure to Brucella. ”*

Others changes can be visualized in the new Fig.1 and Table.1.

**In brucellosis endemic countries, as occupational exposure is always one of the most important risk factors, infections with wildtype Brucella are very common; which means the authors’ argument that sero-positivity is due to Brucella vaccine is flawed, though some or a few of them can be truly due to the exposure to the vaccine strain.**

**Answer**: We agree with the reviewer and appreciate his comments. Therefore the "RESULTS" section has been redrawed, adding the diagnostic criteria used in each study so that the reader can judge the solidity of the diagnosis.

**Change in the manuscript:**

ABSTRACT (lines 38-40): *“Besides, despite strict protection measures, a percentage of vaccine manufacturing workers developed positive Brucella serology without clinical infection.”*

RESULTS (lines 190-196): *“Although serology does not allow to distinguish between vaccine and wild strains, the possibility of contact with Brucella outside the factory was minimal, given the almost absence of cases in the region where the factory is located: <0.08 cases per 100,000 inhabitants / year in the local official registry (https://www.sergas.es/Saude-publica/Documents/105/BEG_XXV-1.pdf). The fact that none of the workers studied developed the disease could be explained by the lower virulence of both vaccine strains and / or by a smaller bacterial inoculum due to strict compliance with safety measures.”*

RESULTS (lines 219-221): *“ The other 4 case reports had negative cultures and were diagnosed by clinical and serological criteria after accidental punctures with the vaccine Brucella vaccine strains S19 [27,28], Rev.1 [29] and RB51 [30].”*

RESULTS (lines 233-239): *“Nineteen of them cited local or systemic symptoms, while 7 reported no adverse events associated with the accidental exposure. Only one of the veterinarians showed a positive culture, which was taken from the cutaneous injection site. Since current standard serologic assays cannot detect antibodies against RB51 infection [12], and the passive surveillance registry probably underestimates rates of needlestick injuries, the authors concluded that we cannot yet determine whether the RB51vaccine has the potential to cause systemic brucellosis in humans.”*

RESULTS (lines 244-250): *“Gunes et al. published in 2013 a serosurveillance study of 46 veterinary staff assigned to a sheep vaccination campaign in Turkey using the Rev.1 strain, in 2013 [35]. Ten persons became seropositive (Rose Bengal test and Wright test ≥1/160), but only 2 developed symptoms of infection and were treated with antibiotics. At 6 months, all of them showed negative serology (Wright test). The study suffers from some limitations since serologic tests were not performed prior to the vaccination campaign, and the prevention measures applied were not stated.”*

RESULTS (lines 251-256): *“Between 2015 and 2016, Proch et al conducted a study aimed to identify risk factors associated with occupational Brucella infection in 296 veterinary personnel in India [36]. Blood samples were taken from 279 individuals and the Rose Bengal, standard tube agglutination (STAT) and ELISA tests were performed. Previous Brucella needlestick injury with the S19 strain was reported in 12 individuals, of whom 5 had a positive serologic test for Brucella.”*

RESULTS (lines 270-274): *“In 2017, an outbreak of brucellosis caused by S2 strain was reported in Tianzhu County, located in the Gansu province of China, during an animal vaccination campaign [37]. A total of 206 controllers participated in the immunization, of wich 51 were postive by serologic testing (infection rate: 24.8%). Although blood cultures of the 51 workers were negative, 48/51 (94%) suffered fatigue and sweat, 4 had fever, and 5 swelling of the testis.”*

**The study lacked the investigation into the quantitative information on the actual use (relative usage) of different types of the vaccine and the cases. The authors argue that Rev1 is the most virulent vaccine but the quantitative or qualitative evidence is not enough and the statement is not convincing.**

**Answer**: We thank the reviewer for his timely observation. The heterogeneity of the few studies and isolated cases published did not allow us to quantitatively estimate the attack rate of each vaccine strain through meta-analysis, as the reviewer rightly points out. The claim of the greater virulence of the Rev.1 strain for humans is based on Spink's experimental study (Reference 7) who compared it with the S19 strain. On the other hand, the RB51 strain has been shown to be less dangerous to humans than the S19 strain (Reference 12) and Chinese publications state that the S2 strain is less virulent for humans than A19 strain, very similar to S19 (Reference 6).

**Change in the manuscript:** These citations (Spink 1960 and Kahler 1998) have been added to the article in both the "INTRODUCTION" and the "DISCUSSION" sections.

INTRODUCTION (LINES 74-82): *“The strains currently used in most countries for the control of bovine brucellosis are Brucella abortus S19 and Brucella abortus RB51, while the strain used for small ruminants is Brucella melitensis Rev.1 [5]. In China, Brucella abortus A19, a strain derived from S19, is used for cattle, and Brucella abortus S2 strain for pigs [6]. These strains are capable of establishing limited infection in livestock, mimicking the natural infection process by wild strains and thus conferring protection. However, these vaccines are not used in humans due to the high risk of developing acute brucellosis [4,7]; they are capable of infecting humans with occupational exposure via the oral, nasal, or conjunctival or genital routes, and by accidental needle inoculation [8,9].”*

DISCUSSION (lines 303-309): *“The danger of these Brucella vaccine strains for use in human inoculation has been studied in various clinical trials. In 1962 Spink et al. carried out a clinical trial of the Brucella vaccines conducted in Minnesota, USA [7]; 11 (67%) of the 16 volunteers receiving the Rev.1 strain developed acute brucellosis, four of them requiring hospitalization, while only 4 (25%) of the 16 individuals receiving the S19 vaccine reported “undesirable sequelae”. The efficacy and safety of human vaccination with Brucella attenuate strains, mainly S19, has been studied in some population studies in the last 60 years.”*

**The suitability of this article to PLoS NTD should be judged by the editor, but I am not convinced due to above reasons. However, the information on the risk of brucellosis vaccine to humans is still indeed scarce. I would like to encourage the authors to anyway improve the article to make it more informative.**

**Answer**: We hope that the article has been suficiently improved after following the instructions of the reviewers.

**Specific comments**

**Material and methods
Line 69: Please Italicize “Brucella”.**

**Answer:** Done.
 **Results
Line 75-76: The sentence is not clear: “Eleven of these articles were identified by manual search of the references found in the full text articles reviewed, and one of them was included in the study”. Out of 12, 11 were found from manual search and 1 was by what? Manual search is also not clear.**

**Answer**: We agree with the revier that our sentence was not clear enough and appreciate his comments.

**Change in the manuscript:** The first paragraph of the article has been changed.

RESULTS (lines 119-122): *“The PRISMA Flow Diagram is shown in Fig 1. Nineteen articles were included from 185 records screened. Of the articles finally included, 12 were identified with the first algorithm, 3 with the second algorithm, 1 with the third algorithm, and 3 more from review of the reference lists. Table 1 lists the publications of brucellosis cases acquired through laboral exposure to vaccine strains.”*

**Line 108: “Since current standard serologic assays cannot detect antibodies against RB51 infection”, please provide the reference here. What about animals?**

**Answer:** An article that discusses the RB51 strain in depth has been included: Reference 12. **Line 146: RB51?**

**Answer:** Thank you. In fact, it is the RB51 strain. The mistake has been corrected. **Line 158: Having brucellosis – having had brucellosis, or was the prevalence 4.6%?**

**Answer: Thank you.** It has been corrected by changing "*having*" to "*having had*".
 **Line 169: Sero-positivity associated with higher levels of exposure to the vaccine strains: what is the level of exposure you mean? High level is the high degree of invasion?**

**Answer:** We agree with the inaccuracy of the expression. "Higher level of exposure" refers to workers who had close contact with biological products and aerosols.

**Change in the manuscript:** The expression has been changed to *“greater level of proximity to Brucella vaccine strain cultures”.* **Discussion
Line 177: How did you judge that Rev.1 seems to be the most virulent?**

**Answer:** The claim of the greater virulence of the Rev.1 strain for humans is based on Pink's experimental study (Spink 1962, Reference 7), who compared it with the S19 strain. On the other hand, the RB51 strain has been shown to be less dangerous to humans than the S19 strain (Kahler 1998, Reference 12) and Chinese publications state that the S2 strain is less virulent for humans than A19 strain, very similar to S19 (Zhou 2020, Reference 6).

**Change in the manuscript:** These citations (Spink 1960, Kahler 1998, Zhou 2020) have been added to the article in both the "INTRODUCTION" and the "DISCUSSION" sections.

**Line 183: Please use references to state about the incidence in manufacturing workers.**

**Answer:** Following the reviewer's instructions, these referencies have been included in the first paragraph of the “DISCUSSION” section, as well as in the “RESULTS” section.

**Change in the manuscript** (lines 291-295):

*“Of the 5 vaccine strains currently used (Rev.1, S19, RB51, A19 and S2), Rev.1 seems to be the most virulent [6,7,12]. Prolonged contact with the pathogen, lack of information and instructions provided to the occupational groups exposed, and low adherence to personal protective equipment (PPE) in the work environment, appeared to be the main risk factors leading to infection by these vaccine strains [24,36,37,38].”* **Line 190: which country Spink conducted the clinical trial?**

**Answer:** We apologize for the lack. The phrase has been changed, inserting *“conducted in Minnesota, USA, in 1962”.*

**Change in the manuscript** (lines 303-307):

*“The danger of these Brucella vaccine strains for use in human inoculation has been studied in various clinical trials. In 1962 Spink et al. carried out a clinical trial of the Brucella vaccines conducted in Minnesota, USA [7]; 11 (67%) of the 16 volunteers receiving the Rev.1 strain developed acute brucellosis, four of them requiring hospitalization, while only 4 (25%) of the 16 individuals receiving the S19 vaccine reported “undesirable sequelae”.”* **Line 199: clinical trial in Greece – it was considered safe enough: please discuss why it was considered so by the authors at that time.**

**Answer:** The authors only reported local inflammation or transient systemic symptoms. Following the reviewer's instructions, we have briefly discuss why it was considered safe enough.

**Change in the manuscript** (lines 317-320):

*“Lastly, in 1994, Hadjichristodoulou et al. conducted a clinical trial on 271 volunteers in Greece; although the S19 vaccine caused some side effects in a quarter of subjects, specifically local inflammation and flu-like symptoms, it was considered safe enough for use on a large scale [42].”* **Line 230: Please provide the key findings clearly when you refer systematic reviews. What were the risk factors for example? Exposure to vaccine strains through vaccination to animals?**

**Answer:** Following the reviewer's instructions, we have modified the manuscript, highlighting the lack of information found in these systematic reviews regarding the risk to humans from *Brucella* vaccine strains. Furthermore, all the reports collected by their authors are already included and discussed in detail in our manuscript.

**Change in the manuscript**: The reference to systematic reviews has been redrafted and, in addition, the section has been changed, discussing them in the "INTRODUCTION" section.

INTRODUCTION (lines 89-99): *“Two meta-analyses on the occupational risks of contracting brucellosis have been published, with some references to the risks that Brucella vaccine handling poses for humans. One of these, by Xie et al (2018), analyzed 27 papers reviewing adverse effects in animals (n = 23) and humans (n = 4) associated with three licensed brucellosis vaccines: S19, Rev.1 and RB51 [14]. They found that human adverse effects from occupational exposure to the vaccines usually involved behavioral and neurological systems, and highlighted that no fatal or permanent human damage was reported. The other systematic review was published by Pereira et al. in 2020 [15], addressing the risk for human of contracting brucellosis by savage and vaccine Brucella strains. Regarding Brucella vaccine strains, they found only 7 elegible reports unsuitable for conducting a meta-analysis. New evidence has recently been published that sheds light on the risk of infection to humans by these vaccine strains.”*

**Line 239-241: Enormous number of vaccine doses administered to humans? In which countries? Please present the evidence.**

**Answer:** Following the reviewer's instructions, we have searched and found a clear and concise reference to the enormous dimensions of the *Brucella* vaccine market: the global brucellosis vaccines market size is estimated to be valued at US$ 224.64 Mn in 2022 and expected to exhibit a CAGR of 4.7% between 2022 and 2030, mainly in North America, Europe and Asia Pacific [https://www.coherentmarketinsights.com/market-insight/brucellosis-vaccines-market-5038].

**Change in the manuscript**: This web reference has been added to the text at the beginning of the “DISCUSSION” section.

DISCUSSION (lines 286-291): *"Our results show that there is a risk of occupational infection by Brucella vaccine strains, although the small number of symptomatic infections recorded compared to the enormous number of doses administered [https://www.coherentmarketinsights.com/market-insight/brucellosis-vaccines-market-5038], suggests that most of them are subclinical. Human can be infected through aerosol exposure and by mucosal and non-intact skin contact with live attenuated strains.”*
 **P266: Here please remind which were effective measures.**

**Answer:** Done.

**Change in the manuscript**: We have added the following words: *“that is to say, proper use of PPE, careful handling of vials and needles, and correct disinfection measures”*.

DISCUSSION (lines 383-387): *“Only two of the studies in our analysis [36,38] evaluated which protective measures were the most effective for exposed workers (that is to say, proper use of PPE, careful handling of vials and needles, and correct disinfection measures), but they were not entirely conclusive due to the difficulty in assessing degree of individual adherence to PPE use. ”*

--------------------

PLOS authors have the option to publish the peer review history of their article ([what does this mean?](https://journals.plos.org/plosntds/s/editorial-and-peer-review-process#loc-peer-review-history)). If published, this will include your full peer review and any attached files.


If you choose “no”, your identity will remain anonymous but your review may still be made public.


**Do you want your identity to be public for this peer review?** For information about this choice, including consent withdrawal, please see our [Privacy Policy](https://www.plos.org/privacy-policy).

Reviewer #1: Yes: Jorge C. Wallach

Reviewer #2: No

Reviewer #3: No


Figure Files:
While revising your submission, please upload your figure files to the Preflight Analysis and Conversion Engine (PACE) digital diagnostic tool, https://pacev2.apexcovantage.com. PACE helps ensure that figures meet PLOS requirements. To use PACE, you must first register as a user. Then, login and navigate to the UPLOAD tab, where you will find detailed instructions on how to use the tool. If you encounter any issues or have any questions when using PACE, please email us at figures@plos.org.
 
Data Requirements:
Please note that, as a condition of publication, PLOS' data policy requires that you make available all data used to draw the conclusions outlined in your manuscript. Data must be deposited in an appropriate repository, included within the body of the manuscript, or uploaded as supporting information. This includes all numerical values that were used to generate graphs, histograms etc.. For an example see here: http://www.plosbiology.org/article/info%3Adoi%2F10.1371%2Fjournal.pbio.1001908#s5.
 
Reproducibility:
To enhance the reproducibility of your results, we recommend that you deposit your laboratory protocols in protocols.io, where a protocol can be assigned its own identifier (DOI) such that it can be cited independently in the future. Additionally, PLOS ONE offers an option to publish peer-reviewed clinical study protocols. Read more information on sharing protocols at https://plos.org/protocols?utm_medium=editorial-email&utm_source=authorletters&utm_campaign=protocols

__________________________________________________
In compliance with data protection regulations, you may request that we remove your personal registration details at any time. (Use the following URL: https://www.editorialmanager.com/pntd/login.asp?a=r). Please contact the publication office if you have any questions.
