# Supplementary material for: What risk do Brucella vaccines pose to humans? A systematic review of the scientific literature on occupational exposure
Source: PLoS Negl Trop Dis. 2024 Jan 8;18(1):e0011889. doi: 10.1371/journal.pntd.0011889 (PMC10830018; doi:10.1371/journal.pntd.0011889)
Supplement: Attachment — Submitted filename: PNTD-D-23-00623R1 Response to Reviewers.doc [file pntd.0011889.s003.doc]

**Responses to review comments** (For ease of review, the changes have been underlined).

**Reviewer #3:**

**Abstract**

**Line 40: risk factors – please rephrase unless statistics are conducted. For example, commonly associated with** …

**Answer**: We agree with the reviewer and the expression has been corrected.

**Change in the manuscript:**

Lines 39-41: *“Prolonged contact with the pathogen, lack of information and a low adherence to personal protective equipment (PPE) use in the work environment were commonly associated with infection.”*

**Line 44: separate ‘workersappeared’.**

**Answer**: We regret the error and the word has been separated.

**Change in the manuscript:**

Lines 44-45: *“Recommended protective measures and active surveillance of exposed workers appeared to reduce this risk.”*

**Introduction**

**Line 72: After the first appearance of Brucella, it can be abbreviated as B.**

**Answer**: We thank the reviewer for this observation. Certainly, this change speeds up the reading of the text.

**Change in the manuscript:**

Lines 71-74: *“The strains currently used in most countries for the control of bovine brucellosis are Brucella abortus S19 and* B. abortus *RB51, while the strain used for small ruminants is B. melitensis Rev.1 [5]. In China, B. abortus A19, a strain derived from S19, is used for cattle, and B. abortus S2 strain for pigs [6]”*

**Results**

**Line 153, please show the date or month of the accident. In the line 157, it says ‘until November’, and readers would want to know how long after the accident the investigation was carried out.**

**Answer**: We find this observation important and the text has been briefly modified to better specify the chronology of the event.

**Change in the manuscript:**

Lines 153-163: *“During late July to August 2019, a laboratory accident with dramatic public health consequences occurred at a Brucella vaccine factory in the city of Lanzhou, located in Gansu province of northwest China. The outbreak initially affected 213 individuals from the nearby Veterinary Research Institute, 8 workers from the biopharmaceutical plant, 2,500 residents of neighboring areas, and 150 people located further away [19]. Until November 30, 2020, when the investigation was completed, the infection was confirmed in 10,528 individuals after testing 68,571 people (attack rate 15.4%) [20]. There were no deaths reported related to this outbreak. Samples from the outbreak patients were subsequently analyzed using a specific PCR that identified the A19 strain, providing pathogenic evidence of the vaccine-derived infection outbreak [21]. Transmission appears to have been by aerosols spread by the wind in a southeast direction, with some evidence of zoonotic transmission.”*

**Lines 154, 195, 215 and so forth: Please Italicize Brucella.**

**Answer**: We apologize for this typographical error. The text has been carefully revised and corrected in 7 places.

**Change in the manuscript:**

We have had to italize *Brucella* in the current lines numbers: 194, 196, 216, 247, 249, 251 and 267,

**Line 196: one digit below zero – please unify this format throughout the manuscript.**

**Answer**: We thank the reviewer for his advice. All text has been revised and percentage values have been plotted to 1 decimal.

**Change in the manuscript:**

You can check these changes on the lines: 185, 197, 220, 237, 253, 259, 265, 289, 290, 296, 299, 335 and 336.

**Line 196: It should be ‘sero-positive rate’ or ‘sero-prevalence’, instead ‘infection rate’.**

**Answer**: We agree with the reviewer that the new expression is more precise and it has been changed in place of the previous one.

**Change in the manuscript:**

Lines 196-198: *“They reported a sero-prevalence of 43.6% (61/140), although all workers were asymptomatic and no suspected or confirmed case was found [24].*

--------------------

We sincerely thank the reviewer for the great effort made to correct our manuscript and we hope that we have adequately responded to all his comments. We believe that in this way the paper has been significantly improved.
